# Effect of Different Agricultural Farming Practices on Microbial Biomass and Enzyme Activities of Celery Growing Field Soil

**DOI:** 10.3390/ijerph182312862

**Published:** 2021-12-06

**Authors:** Lin Wang, Mandeep Kaur, Ping Zhang, Ji Li, Ming Xu

**Affiliations:** 1 Henan Key Laboratory of Earth System Observation and Modeling, College of Geography and Environmental Science, Henan University, Kaifeng 475004, China; wanglin@henu.edu.cn (L.W.); mk9041985@gmail.com (M.K.); 18203635580@163.com (P.Z.); 2 Department of Environmental Sciences, Miami College, Henan University, Kaifeng 475004, China; 3 College of Resources and Environmental Sciences, China Agricultural University, Beijing 100193, China

**Keywords:** agriculture, conventional farming, low-input farming, organic farming, soil enzyme, catalase, protease, urease

## Abstract

Soil quality is directly affected by alterations in its microbiological, biological, biochemical, physical, and chemical aspects. The microbiological activities of soil can affect soil fertility and plant growth because it can speed up the cycling of nutrients, enzymes, and hormones that are needed by plants for proper growth and development. The use of different agricultural management practices can influence microbial biomass and enzyme activities by altering soil microclimate, soil microorganism habitat, and nutrient cycling. Based on this, the present work planned to evaluate the impact of conventional, low-input, and organic farming systems in a vegetable field growing celery on microbial biomass and different soil enzyme activities. The present study showed a comparison of the effect of different practices on biological soil quality indicators during two sampling times, i.e., one month after colonization and one month before harvesting. It was observed that the soil microbial biomass in the organic farming system was significantly higher than that found in conventional and low-input practices. Under an organic farming system, the soil microbial biomass in December was significantly higher than that in October. The soil microbial biomass carbon in the 0–20 cm soil layer showed higher variation compared to that in the 20–40 cm layer for all the three of the farming management practices that were used in the study. Additionally, the soil total carbon and total organic carbon were recorded as being higher in the December samples than they were in the October samples. Under all the three of the management practices that were applied, the soil catalase activity was higher in the October samples than it was in the December soil samples that were collected the from 20–40 cm soil layer compared to those that were taken from the 0–20 cm layer. The application of organic fertilizer (chicken and cowmanure compost) resulted inincreases in the soil urease and in the protease activity. The protease activity of the soil samples that were extracted from the 0–20 cm and 20–40 cm soil layers in October was higher in the samples that were taken from farms using conventional practices than it was in the samples that were taken from farms using organic and low-input practices, while the samples that were collected during December from both of the soil layers showed higher protease activity when organic methods had been used. No significant variation in the soil urease activity was observed between the two soil layer samples. Urease activity was the highest when organic management practices were being used, followed by the low-input and the conventional modes. For the conventional and low-input practices, the soil urease activity showed an obvious trend of change that was related to thetime of sampling, i.e., activity in December was significantly higher than activity in October. The novelty of this study was to determine the microbial biomass carbon and enzymatic activity in a six-field crop rotation (tomato, cucumber, celery, fennel, cauliflower, and eggplant) using three management practices: low-input, conventional, and organic systems. The present study showed that the long-term application of organic fertilizers plays a large role in maintaining excellent microbial and enzyme activitythat result in improved soil quality.

## 1. Introduction

Agriculture represents a critical sector of world’s economy. Sustainable land management can increase the agricultural production and can reduce losses in biological diversity. In land management, one of the main challenges is to conserve the soil quality as well as the associated ecosystem services while increasing the agricultural yields [1]. Soil quality represents the potential of a soil to work well within various ecosystem boundaries in order to preserve biological productivity, maintain environmental quality, and promote plant growth and development [2]. Land management techniques like conventional, low-input etc. can result in variations in soil organic matter following loss of the organic carbon and ultimately affect the microbial biomass of particular soil ecosystem [3]. Conventional farming has generally led to a decline in soil structure and soil aggregation, which results in reduced water infiltration, increased soil bulk density, and salinity and nitrogen leaching, all of which contribute to ground water contamination [4,5]. Compared to conventional land management techniques, organic farming results in better food quality and safety due to its biological nutrient supply and pest control methods [6]. Under different crop management practices, organic farming can lead to higher soil quality than conventional farming due to enhanced soil biodiversity [7], better soil structure formation [8], and increased enzyme activity, microbial biomass, and soil organic carbon [9,10]. Organic farming represents one of the most important and holistic production management systems and is able to promote agro-ecosystem health by improving biological cycles, biodiversity, soil biological activity, etc. This type of crop management practice avoids or excludes the use of synthetic compounds, plant growth regulators, genetically modified organisms, fertilizers, pesticides, livestock food additives, etc. Organic farming encourages the use of crop residues, animal manures, plant nutrients (rock phosphate, basic slag, rock potash), legumes, green manure (cowpea, green gram), off-farm organic waste, biofertilizers (*Rhizobium*, *Azotobacter*, *Azospirillum, Rhizobacter*, Mycorrihizal fungi, Blue green algae, Azolla), etc., as well as the use crop rotation, mechanical cultivation, and biological pest (insects, weeds) control to maintain the overall soil productivity [11]. Organic farming is able to help maintain the health of the environment, reduces human and animal hazards, keeps agricultural production at a sustainable level, reduces the cost of agricultural production, results in the optimumuse of natural resources; improves soil physical properties such asgood tilth, granulation, good aeration, and high water holding capacity; and reduces soil erosion [6]. Since 1985, organic farming and the production of organic food has continued to grow across the world. By 2017, a total of 69.8 million hectares land were dedicated to organic farming globally, representing 20% growth compared to the year 2016 [12]. Among various countries, Australia has the largest area that is dedicated to organic agricultural land (35.65 million hectares), followed by Argentina, China, Spain, the USA, Italy, India, etc., while India has highest number of organic producers in the world, followed by Uganda, Mexico, Ethiopia, the Philippines, etc. [12]. Organic farming is practiced by 187 countries around the world and covers about 72.3 million hectares of agricultural land [13].

The European Union recently launched the Farm to Fork (F2F) strategy under the “European Green Deal”, which includes a set of different policies that have been determined by the European commission that aim to introduce new legislation on organic farming and food security. The European Commission’s F2F strategy initiated various initiatives, with aim of at least 25% of the agricultural land in the European Union agricultural land being managed by organic farming methods by 2030. Additionally, the F2F future targets included a 50% reduction in pesticide use o; a 20% reduction in fertilizer use; a 50% reduction in nutrient loss; a 50% reduction antimicrobial use in agriculture and aquaculture activities; a 50% reduction in food waste; and sustainable food labeling by 2030 [14,15,16].

Different chemical and microbiological parameters can be used as indicators of soil quality [17,18]. Bell and Raczkowski [19] observed that the physical, chemical, and biological soil quality indicators can reveal rapid changes in soil conditions that are the result of different soil management methods. Qin et al. [20] recommended that the microbial biomass nitrogen (MBN), MBC, and activities of different enzymes were found to be the most effective indicators for monitoring soil quality and productivity. MBC represents the microbial population and that acts as in indication of organic carbon turnover in soil, while soil enzymes act as important constituents that catalyze the reactions that are important for organic matter decomposition and for the cycling of nutrients [21,22,23,24]. Urease enzyme activity is important for nitrogen cycling, while phosphorus cycling depends on phosphatase enzyme activity [25,26]. No-till farming can increase the amount of organic matter that is able to enter the soil and reduces soil disturbances and erosion; it also favors MBC and different enzyme activities [27,28]. Many authors have reported that the microbial population and enzyme activities in soil are generally higher in organically managed farming soils compared to in conventional and other farming practices [29,30]. Soils can also be classified according to soil enzyme activity, allowing the productivity level of agricultural soil to be evaluated [31]. The level of soil enzyme activity can directly affect the soil material transformation and circulation rate and can quickly respond to alterations that have been induced by external factors such aspH, water content, and soil temperature [32].

Presently, there is a growing concern among farmers and researchers to adopt alternative farming practices that can improve thebiological properties of soil and that can thereby sustain good soil health and high crop productivity. During the last many years, several studies comparing the abundance of microbial communities and enzyme activities insoil under different management systems (organically and conventionally) have been published. However, there are less studies that have provided knowledge about the long-term effects of organic farming applications on the microbial parameters that are oftenreported upon. Keeping all of this in mind, it was determined that the present study would compare soils from three greenhouses that were operating under three different agriculture practices (conventional, low-input, and organic farming systems) thatwere likely to show differences in terms of the microbial biomass carbon and soil enzyme activityat the Quzhou Experimental Station of the China Agricultural University, China. The objective of present study was to estimate the long-term effects of different agricultural practices on the microbial biomass carbon and the soil enzyme activity in soil being managed through different agricultural practices.

## 2. Materials and Methods

### 2.1. Field Experiment

This experiment highlighting the effects of long-term management practices has been being conducted at the Quzhou Experimental Station of China Agricultural University in the North China Plain (36°52′ N, 115°01′ E) since 2002. The station is warm and has asemi-humid climate thatcomprises dry and cold winters and rainy summers. The mean annual temperature is 13.2 °C, and the soil type is an improved silt fluvo-aquic soil [33]. The initial basic soil properties at the 0–20 cm and 20–40 cm depths are shown in Table 1.

Three greenhouse farming systems were selected for the present experiment and included those using conventional management practices (CON), low-input management practices (LOW), and organic management practices (ORG). Each greenhouse is 52 m in length and 7 m in width. Tomato, cucumber, celery, fennel, cauliflower, and eggplant were grown in rotation (mainly tomato and cucumber) and were planted in the fields, and two or three types of vegetables were planted annually. The conventional system used chemical fertilizers, pesticides, and chickenand cowmanure produced for the local style of greenhouse vegetable production. For the low-input system, the fields received 50% chemical fertilizer and 50% compost fertilizer and used biological and low-toxic chemical pesticides for plant protection. The organic management system was conducted based on the International Federation of Organic Agriculture Movements (IFOAM) and used chicken and cowmanure compost and biological and physical methods to protect the plants.

### 2.2. Soil Sampling

The study area for the present work was a celery-growing field, and soil samples were taken from the study area on two different occasions(October and December): one month after colonization and one month before harvesting/maturity for the year of 2016. The three greenhouses weredivided into four samples, with each sample being taken according to the model S and were taken using the 5-point sampling method. Soil samples with four replicates per system were taken at the 0–20 cm and 20–40 cm depths. After the soil had been appropriately dried by the sun, the soil samples were each placed into aseptic bags, labeled, and kept in the labrefrigerator 4 °C until they were able to be processed as soon as possible.

### 2.3. Analysis of Soil Microbial Biomass and Soil Enzyme Activities

Soil microbial biomass carbon (MBC) was determined by the chloroform fumigation extraction method [34]. About 20 gof fresh soil samples (passed through 2 mm sieve) was placed into vacuum dryers with a layer of wet filter paper and was fumigated with chloroform using a vacuum pump (25 °C; for 48 h in the dark wrapped with black cloth). Further, asoil sample in abeaker was transferred to aconcussion bottle containing 60 mL of 0.5 M potassium sulfate. The soil sample was centrifuged at 3000 rpm/min for 15 min. About 5 mL of the filtrate was placed into the liquid storage bottle and was further analyzed by a TOC analyzer (elementar, liquid TOC II, Jena, Germany).

#### 2.3.1. Soil Enzymes Activity

The activity of the enzymes that are commonly found in soil, such asurease, catalase, and protease, was also determined. Urease activity was measured spectrophotometrically using urea as a substrate, while catalase and protease activity was measured by employing the volumetric method and the ninhydrin colorimetric method, respectively [35].

#### 2.3.2. Catalase Activity

To determine thecatalase activity, 5 g of a dry soil sample was put into a 150 mL triangle flask, to which 40 mL of distilled water and 5 mL of 0.3% H_2_O_2_ solution were injected. After shaking the triangle flask (120 r/min) for 30 min, about 5 mL of 1.5 mol/L sulfuric acid was added and centrifuged (3000 rpm/min for 15 min). The control sample was also prepared the same way without a soil sample. About 25 mL of filtrate was titrated against 0.002 mol/L KMnO₄ until a light pink colour was obtained as an end point. The catalase activity (hydrogen peroxide enzyme) was expressed as 0.002 mol/L potassium permanganate ml per unit of soil weight consumption (difference between control and experimental determination). The soil hydrogen peroxide enzyme activity(ml KMnO4/gdry soil)wascalculated by formula:

Soil hydrogen peroxide activity = V / dwt

where, V is 0.002mol/L potassium permanganate mL (mL); dwt is the weight of the drying soil (g).

#### 2.3.3. Protease Activity

Protease activity was determined by taking 2 g of dry soil sample in a triangular flask, to which 0.5 mL toluene was added, mixed, and leftundisturbed for 15 min. Further, about 10mL of 1% white gelatine was added to the soil sample, and the sample was then shaken well. After shaking, the triangular flask was cultured in a water bath at 30 °C for 24 h before the sample was centrifuged (3000 rpm/min for 15 min) and filtered. An amount of 5 mL of filtrate was absorbed into a test tube, and 0.5 mL of 0.05 mol/L H_2_SO_4_ and 3 mL of 20% Na_2_SO_4_ were added to precipitate the protein, and the sample was filtered again. From the solution, about 2 mL of filtrate was put into a test tube, to which 1 mL of 2% nin-hydrin solution was added, and test tube was heated in a boiling water bath for 10 min. After boiling, the solution was diluted to 50 mL with distilled water. Additionally, the control sample was prepared using the above process without the addition of the soil sample. Protease activity was determined spectro-photometerically (Shimadzu, UV-3600, Kumamoto, Japan) by measuring the wavelength at 560 nm. The protease activity of the soil samples was calculated by using the standard curve forglycine. The activity of the soil-hydrolyzed protease enzymes wasexpressed as the micrograms of glycine in a1gsoil sample after 24 h of treatment.

Glycine (μg,g^−1^) = (c × 50 × ts) / m

where glycine (μg,g^−1^) is the amount of micrograms of glycine in a1g soil sample after 24 h; c—glycine concentration found on the standard curve, μg, mL^−1^; 50—colored liquid product (mL); ts—multiple (here is 2 s 10/5); m—soil mass (g).

#### 2.3.4. Urease Activity

For the urease activity, 20 g of dry soil samples were placed into a 100 mL volumetric flask and were treated with 2 mL toluene for 15 min. The volumetric flask was placed in an incubator for 24 h at 37 °C. The samples were further transferred to capacity bottles, to which 10 mL of 10% urea solution and 20 mL citric acid buffer (pH 6.7) were added and shaken well. After mixing, the bottles were placed in a temperature box for 24 h at a temperature of 37 °C. Afterwards, the samples were centrifuged (3000 rpm/min for 15 min), and about 3 mL of the supernatant was absorbed into a 50 mL volumetric flask along with 10 mL of distilled water. At the same time, the control sample was also prepared the in same way butwithout the inclusion of a soil sample. The urease activity of the final soil solution was determined using a spectrophotometer (Shimadzu, UV-3600, Kumamoto, Japan) at the 578 nm wavelength. The urease activity of the soil samples was calculatedusing the standard curve forammonium sulfate that has been dissolved in water and diluted to 1000 mL(100 ppm) with 0.1 mg of nitrogen. The enzyme activity in NH3-N represents the number of milligrams of ammonia released by the enzyme deuresis urea per gram of soil.

### 2.4. Statistical Analysis

The data wereanalyzed and processed using Microsoft Excel 2010 software. SPSS19.0 software was used for the statistical analyses. The significance of the results was examined with one-way analysis of variance (ANOVA) with the Duncan test, and differences were considered significant if *p* < 0.05.

## 3. Results and Discussion

### 3.1. Microbial Biomass Carbon

**Figure 1:**
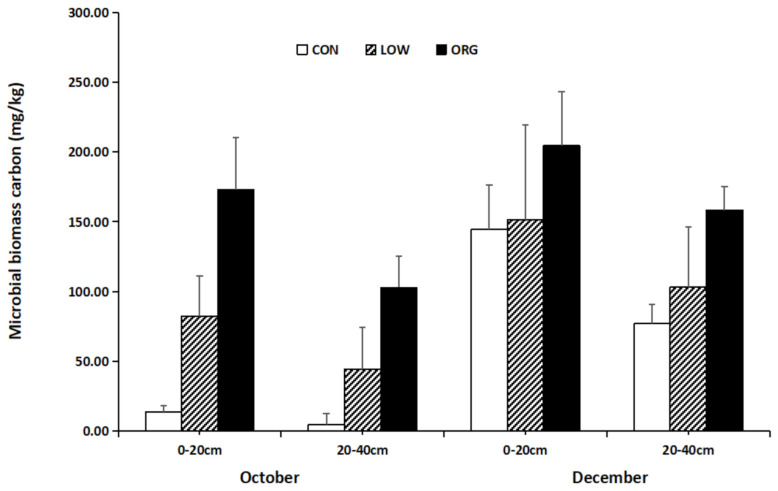
Effect of conventional, low-input, and organic practices on soil microbial biomass carbon.

The soil microbial biomass is the source and reservoir of plant nutrients and actively participates in the nutrient cycle [36]. It represents the active part of soil nutrients, so it is often used to evaluate the biological characteristics of soil quality. Based on analysis, it was found that the long-term use of organic farming practices involving the application of composting fertilizers to the soil significantly increased the microbial biomass carbon (Figure 1). As shown in Figure 1, the soil microbial biomass carbon in the soil being cultivated using organic practices was the highest, followed by the content that was obtained in the soil being cultivated by the low-input practices, and the lowest was observed in the soil that was being cultivated by conventional practices. The variation in the soil microbial biomass carbon in the 0–20 cm soil layer was higher than that in the 20–40 cm soil layer for all three of the farming practices.

For the sample soils that were taken during the October sampling period, the samples that had been taken from the low-input practice greenhouse at both the 0–20cm and 20–40cm soil layers showed a soil microbial biomass carbon level that was 4.89 times and 11.36 times higher, respectively, than those obtained from the samples from the greenhouse using conventional practices. For the soil samples that were taken from the greenhouse using organic practices, the amount of microbial biomass carbon was 8.59 times and 21.27 times higher than the amounts that were achieved at both depths in the soil samples that were taken from the greenhouse using conventional modes of cultivation. The soil total carbon and total organic carbon in the same soil layer were sampled twice and were higher in December than they were in October.

Some studies have shown that soil microbial biomass can quickly respond to variations in different fertilization practices, crop systems, and land use patterns [37,38,39]. Soil microbial carbon content acts as a significant contributor tosoil productivity and can control soil nitrogen (N) availability and the regulation of the physico-chemical properties of soil [40]. Fertilization management has shown that the application of organic fertilizer can significantly improve the soil microbial biomass carbon content and basal respiration intensity, which was evident in the present study and was consistent with the findings of other studies [41,42,43,44,45]. A high MBC can indicate an increase in N storage in soil. Singh et al. [46] reported a significant increase in soil microbial biomass carbon and basal respiration in rice–wheat and rice–wheat mungbean cropping systems in the Indo-Gangetic plains under organic farming conditions involving the combination of vermincompost, crop residues, and bio-fertilizers.

### 3.2. Soil Enzymatic Activities

Soil enzymatic activity may be influenced by different agronomic farming practices such as organic or conventional farming; organic systems have been shown to have asignificant impact on the soil enzyme activity [47,48,49].

#### 3.2.1. Soil Catalase Activity

The catalase enzyme is widely found in soil organisms. It promotes the decomposition of hydrogen peroxide, a free radical that is harmful to terrestrial plants when it is able to enterwater and oxygen; as a result, the catalase enzymeprevents the toxic effect that hydrogen peroxide has on soil enzymes [50]. It acts as an important oxido-reductase system for the synthesis of humus in soil [51]. Catalase plays an important role in soil ecosystem, and it can be used as a biological activity index to evaluate the quality of a particular soil [52]. As such, studies on the variationsthat can occur insoil catalase under different farming practices is of great significance if we are to understand the rational utilization of fertilizers, soil resources, and the construction of sustainable soil ecosystems. Catalase activity depends onmicrobial biomass, organic oxygen concentration, changes in CO_2_, dehydro-genase, glucosidase, and esterase activity in soils. Its high activity predicts high soil fertility and the presence of aerobic micro-organisms in the soil [53].

**Figure 2:**
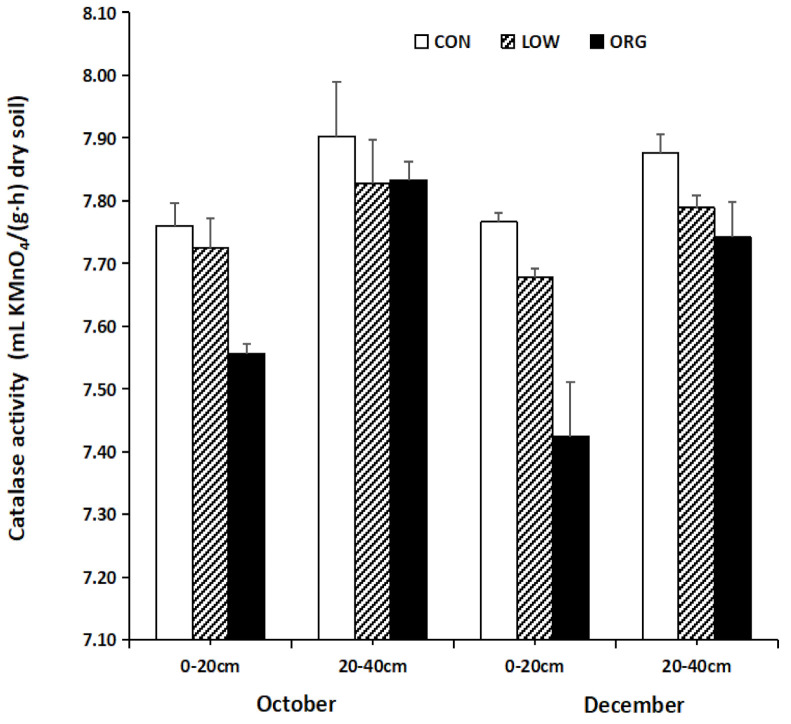
Effect of conventional, low-input, and organic practices on soil catalase activity.

The catalase activity (Figure 2) in the 20–40 cm soil layer was higher than that in the 0–20 cm soil layer under all three practices. The catalase activity in October in the same soil layer under the same practice was higher than that in December. The catalase activity in October and December had the same response order to different management modes, whereas the conventional mode showed high catalase activity compared to the low-input and organic farming modes. Kobeirski et al. [54] reported significant high catalase activity in soil that had been cultivated under organic farming practices compared to soil that had been cultivated using conventional practices. Additionally, Filipek-Mazur et al. [55] reported high catalase activity in the stagnic luvisol soil type under organic farming practices and concluded that the enzymatic activity of soil depends on the type of soil and the variety of crops that are grown in that soil. Contradictory to the present results, Purev et al. [51] observed high catalase activity at the 0–15 cm depth in mountainous, dry steppe, and humidified soils while activity decreased rapidly as the depth increased.

#### 3.2.2. Soil Protease Activity

**Figure 3:**
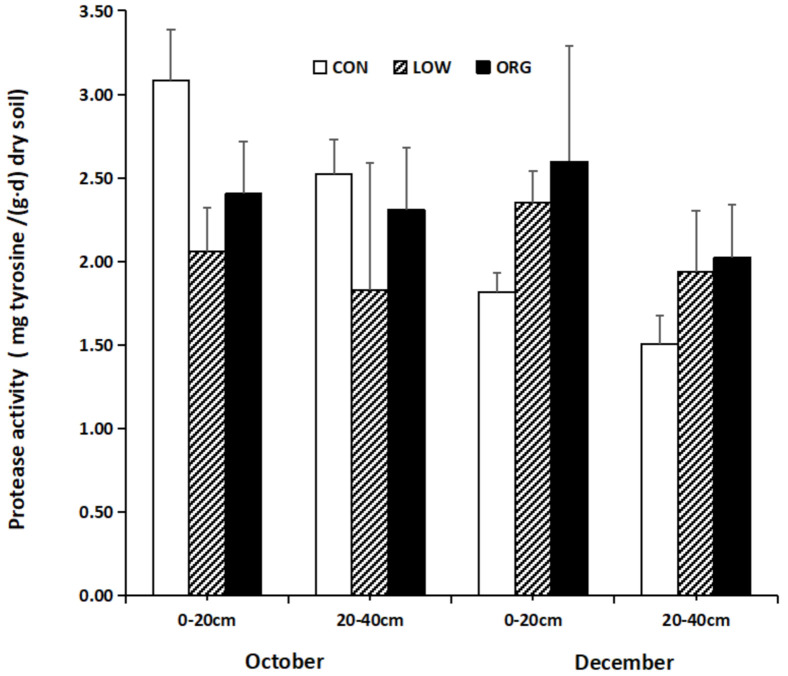
Effect of conventional, low-input, and organic practices on soil protease activity.

Protease is a large type of enzyme that exists at high concentrations in soil, and it can hydrolyze various proteins and peptides and other compounds into amino acids [56,57]. The protease activity in soil has an important relationship with the transformation of nitrogen nutrition in soil, and it is also an important index that can be used to evaluate the soil fertility level [58]. Soil protease activity is generally higher in soil that is under crop rotation compared to in monoculture soil [59]. Protease activity indicates the nitrogen mineralizationcapacity by the microbial communities that are present in the soil. From Figure 3, it can be observed that the protease activity in the 0–20 cm soil layer in the three different practices is higher than that in the 20–40 cm soil layer. However, the soil protease activity in October and December was not consistent in response to the different practices. In October, the protease activity in the 0–20 cm and 20–40 cm soil layers was higher in the conventional practice soil samples than it was in the organic and low-input practice samples. However, in December, the protease activity in both of the soil layers was higher in the soil samples that were taken from the greenhouse using organic practices compared those taken from the greenhouses using low-input and conventional modes of cultivation. In the conventional practice samples, the soil proteinase activity in October was higher than thatfrom the same soil later in December, and in low-input mode, the proteinase activity in December was higher than that from the same soil layer in October.

Similar to the obtained results, Purev et al. [51] reported the highest protease activity in the layer that was 0–15 cm from the soil surface and observed that the protease activity rapidly declinedas the soil depth increased. The protease activity sharply increased in the soil that had been cultivated under organic farming practices compared to in the soil that had been cultivated under conventional farming practices [60]. Additionally, protease enzyme activity has been shown to be able increase in organic farming systems when the soil pH is favorable and when there is a higher content of total nitrogen, organic carbon, etc. [61], a phenomenon that was also observed in this study. Similarly, Niewiadomska et al. [62] reported thatincreased protease enzyme activityin maize-cultivated soil from adidactic farm at the Department of Soil and Plant Cultivation, Swadzim (University of Life Sciences in Poznan) depended on the type of organic fertilizer and the dose of that fertilizer applied to soil when organic farming methods were being implemented.

#### 3.2.3. Soil Urease Activity

Urease is widely present in the soil ecosystem, and its enzymatic product, ammonia, is the main source of nitrogen for plants [63]. Urease has close relationship with soil nitrogen ability, and it represents the degree of soil nitrogen supply [64]. High urease activity in soil can rapidly hydrolyze externally applied urea to ammonia, which contributes to soil nitrogen losses and covers up deficiencies in the plants [65]. The increase of urease activity in soil is conducive to the stability of high organic nitrogen in the soil to the effective nitrogen transformation. To improve the soil nitrogen supply, the increase of the urease activity shows that using organic fertilizer to improve soil nitrogen transformation has a better effect.

**Figure 4:**
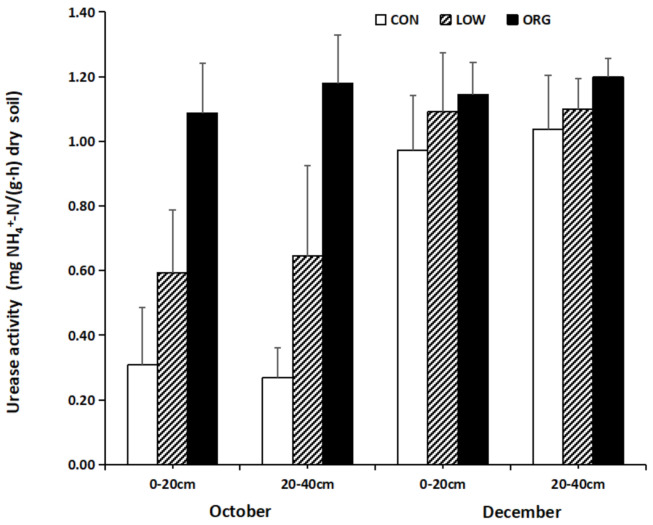
Effect of conventional, low-input, and organic practices on soil urease activity.

The response of the soil urease activity in the conventional, low-input, and organic practices isshown in Figure 4. There was no significant difference in terms of the soil urease activity between the two soil layers, and the changes in the soil urease activity in the same soil layer showed obvious consistency duringboth sampling periods. Under the organic conditions, the urease activity was the highest, followed by the low-input and the conventional modes. For the conventional and low-input practices, the soil urease activity showed an obvious trend of change with time, as the activity in December was significantly higher than that in October. Under organic practices, the soil urease activity showed no obvious changes with time, but slight differences were determined based on the month in which the sample was taken: December and October.Urease activity can be influenced by the presence of organic and inorganic matter content, and it is highly sensitive to heavy metals in the soil [66]. Similar to the present study, Kwiatkowski et al. [61] also observed the highest urease activity in the organically farmed soil, where it was present a higher amount than the soil organic matter. They reported a significant positive correlation between the organic farming of crops such as sugar beet, red clover, and oats and winter wheat and the urease enzyme activity. Additionally, Chen et al. [67] reported a significant increase in the urease activity of tea plantation soil under sustainable (organic) agriculture management practices (after 2 and 25 years of experimentation) across a temporal scale, though no significant differences between different management practices were observed.

## 4. Conclusions

The present study was based on a comparison of the effects of different management practices on soil microbial biomass and soil enzyme activity during two sampling periods, i.e., one month after colonization and one month before harvesting. The present study showed that the soil microbial biomass in the organic farming system was significantly higher in the samples that were collected during December compared to those that were obtained from the soil that had been cultivated using conventional and low-input practices. The soil microbial biomass carbon in the 0–20 cm soil layer showed the highest variation compared to the 20–40 cm layer under all three farming management practices. The soil catalase activity was higher in October than it was in December in the soil samples that had been collected from the 20–40 cm soil layer compared to those that had been collected from the 0–20 cm layer. Under organic farming management practices, the urease, catalase, and protease activity were the highest followed by the low-input and the conventional modes, while samples that had been taken from the 20–40 cm soil layer that were collected during December showed high soil enzymatic activity compared to the samples that had been collected in October. The present work revealed that the long-term application of organic fertilizers can improve the soil quality and can exert a strong positive effect on the abundance of soil microbial communities as well as on the enzyme activity. This study also showed that organic farming can lead to crop yield stabilization and that it can make the crop yield more sustainable by improving the chemical and biological properties of the soil. It can be concluded that encouraging organic farming can build an ecologically, nutritionally, and economically healthy nation in the near future. Government should encourage farmers to use eco-friendly farming practices, such asthe application of perennials and legumes; the extension of crop rotation cropping system; and the application of more organic fertilizers. More and more organic production should be encouraged, which not only promotes the health of consumers of a nation but also the ecological health and the economic growth of any nation. In the future, more extensive research should be conducted using uniform protocols in order to establish the most suitable agricultural management practice, with the intention of not only improving crop yield but that is also able to maintain resilient soil health.

## Figures and Tables

**Table 1:** The initial basic soil properties recorded in the greenhouse experiment.

Treatments	Distance	Organic Matter (g/kg)	Total N (g/kg)	Total P (g/kg)	Available K (mg/kg)	Alkaline Hydrolytic N (mg/kg)	Available P (mg/kg)
CON	0–20 cm	18.93	1.36	2.22	212.83	128.38	163.05
	20–40 cm	8.75	0.74	1.08	135.28	47.66	48.75
LOW	0–20 cm	15.25	1.19	1.24	364.28	95.35	81.68
	20–40 cm	7.13	0.68	0.79	131.18	34.95	39.42
ORG	0–20 cm	16.63	1.17	1.38	257.30	101.28	139.13
	20–40 cm	9.6	0.77	1.04	129.30	40.43	33.03

## Data Availability

Data are within the article.
